# Effect of pretreatment of root dentin surface with cold atmospheric plasma on improving the bond strength of fiber post and resin cement: In vitro study

**DOI:** 10.1002/cre2.744

**Published:** 2023-06-08

**Authors:** Neshatafarin Manouchehri, Safoura Ghodsi, Faezeh Atri, Pegah Sarraf, Dorsa Seyedi, Sara Valizadeh

**Affiliations:** ^1^ Department of Periodontics and Oral Medicine, School of Dentistry University of Michigan Ann Arbor Michigan USA; ^2^ Department of Prosthodontics, School of Dentistry Tehran University of Medical Sciences Tehran Iran; ^3^ Department of Endodontics, School of Dentistry Tehran University of Medical Sciences Tehran Iran; ^4^ Department of Restorative Dentistry, School of Dentistry, Dental Research Center, Dentistry Research Institute Tehran University of Medical Sciences Tehran Iran; ^5^ Department of Oral Biological and Medical Sciences, Faculty of Dentistry University of British Columbia Vancouver British Columbia Canada

**Keywords:** EDTA, fiber post, nonthermal atmospheric pressure plasma, push‐out, resin cements

## Abstract

**Introduction:**

Achieving stable adhesion between fiber post and interradicular dentin is a challenging process in the restoration of endodontically treated teeth. This study was conducted to investigate the effect of surface pretreatment with cold atmospheric plasma (CAP) on improving the bond strength between them.

**Materials and methods:**

Forty‐eight single‐canal mandibular premolars were cut 1 mm above the cementoenamel junction to keep the root length of 14 mm or more. After endodontic treatment and preparation of the post space, the teeth were divided into four groups regarding the pretreatment of dentin surfaces, including normal saline, ethylenediaminetetraacetic acid (EDTA), CAP, and CAP + EDTA groups. The data were analyzed using paired and independent *t*‐test and one‐way analysis of variance and the significance level was set at *p* < .05.

**Results:**

The bond strength was significantly higher in the coronal third than in the apical third in all the groups. Moreover, the bond strength was significantly higher in the CAP + EDTA‐treated group. The bond strength increased significantly in the CAP group compared to the normal saline group. In addition, the bond strength increased significantly in the CAP or EDTA groups compared to the control group. The lowest bond strength belonged to the control group (normal saline).

**Conclusion:**

The surface pretreatment with CAP (alone or in combination with EDTA) played a significant role in improving the bond strength of fiber post and root canal dentin.

## 1 INTRODUCTION

Restoring endodontically treated teeth is one of the most challenging treatments calling for several considerations to guarantee long‐term successful results (Morgano et al., 1994). Researchers recommend placing the post inside the root canal only when the remaining structure is not sufficient for support, retention, or reconstruction (Mannocci et al., 2021). However, the most common problem in restoring the endodontically treated tooth is post debonding from the root dentin (Bakaus et al., 2018). The dowel post is responsible for maintaining the core and integrity of the edges, and protecting the remaining dental tissue (Mayya et al., 2020). Conventional endodontic posts are categorized as custom‐made and prefabricated. The latter could be made of metal, ceramic, and reinforced fiber (Trushkowsky, 2008). Fiber posts are composed of carbon, quartz, silica, zirconia, and glass fibers in a resin basis and have the ability to bond using an adhesive technique. These posts have higher bond strength to dentin compared to metal posts and cause fewer root fractures (Lamichhane et al., 2014). The forces applied to the teeth will be well absorbed by the fiber posts and create less stress than with other post materials (Asmussen et al., 1999). Self‐adhesive resin cements are a relatively new subgroup of resin cements that do not require initial preparation of the tooth structure and are used exclusively in one step, which reduces potential technical errors (Weiser & Behr, 2015; Wingo, 2018). Since fiber posts are passively retained in the root canal, the efficiency of resin cement and the adhesion process plays an important role in the overall clinical performance of restorations (Barcellos et al., 2011).

Different techniques were introduced for dentin surface preparation to facilitate the penetration and absorption of bonding agents (Sahafi et al., 2004). Cold atmospheric plasma (CAP) has attracted special attention as an effective method for surface modification (Sarkar et al., 2018). CAP is a mixture of highly reactive particles that includes electronically excited molecules/atoms, ions, free radical species, and ultraviolet photons. CAP is able to clean the surface material, react with different substrates, and ultimately change the surface properties of the materials (Liu et al., 2016). It has been claimed that plasma surface treatment facilitates the penetration of adhesive resin into the smear layer of the relatively open dentinal tubules, resulting in a thicker hybrid layer and improving the bond performance (Dong et al., 2015). This study was conducted to investigate the effect of CAP surface pretreatment on improving the bond strength of fiber post and root dentin in comparison to ethylenediaminetetraacetic acid (EDTA) and their combination (CAP + EDTA). The null hypothesis proposed that there will be no significant difference in the bond strength of fiber posts cemented with self‐adhesive resin cement in the cervical and apical third of the root canal between all studied groups.

## 2 MATERIALS AND METHODS

The methodology used in this study was based on the findings of Sadeghi Mahounak et al. (2021), who investigated the effect of root dentin pretreatment on the micro‐push‐out bond strength of fiber posts to root canal dentin. In their study, the authors used cold atmospheric argon plasma and EDTA as pretreatment agents and evaluated their effect on bond strength. We utilized the results of their study as a reference to guide our own investigation.

### 2.1 Sampling procedure and specimen preparation

Forty‐eight single‐canal mandibular premolars with a root length of at least 14 mm extracted for periodontal and orthodontic reasons were selected for this study (ethical approval code: IR.TUMS.DENTISTRY.REC.1399.089). The exclusion criteria were decayed curved roots, cracked roots, open apex, and/or a history of previous endodontic treatment. Any soft tissue debris was removed and the teeth were kept in 0.5% chloramine‐T disinfecting solution for 1 week, after which they were placed in distilled water until the time of use (maximum of 3 months) (Sarraf et al., 2019).

The crowns were cut using a diamond disk (Yuanda) 1 mm above the cementoenamel junction (CEJ). The operating length was determined to be 1 mm shorter. Rotary files were used to clean and shape the root canals, starting from rotatory file size F1 and ending with the final rotary file size F3 (EdgeTaper). Also, 5 mL of NaOCl 5.25% (Chloraxid; Cerkamed) irrigation was used for root canal cleaning. To remove the smear layer, a final flush using 5 mL EDTA 17% (Endo‐solution) followed by 5 mL NaOCl 5.25% was performed. The canal was filled with gutta‐percha (Meta Biomed) and resin sealer (AH+; Dentsply) using the cold lateral compaction technique. The access cavity was sealed using Cavit temporary repair material (3M) and eventually specimens were maintained for 1 week at 37°C and 100% relative humidity in an incubator (Thermo Scientific™ Heratherm™; Thermo Fisher Scientific Inc.) (Valizadeh et al., 2021).

### 2.2 Post space preparation and cementing of the posts

The root canal was cleaned of filling material, which was confirmed by radiographic imaging. A drill (white post system; FGM) was used to prepare the post space. The teeth were then divided into the following groups based on the type of surface treatment, and each group included 12 teeth. Group 1 was treated with 10 mL of 0.9% normal saline (Samen Pharmaceutical Co.) for 60 s and dried. Group 2 was treated with flushing 10 mL of 17% EDTA (Cerkamed) for 60 s, washed, and dried. Group 3 was treated with a helium atmospheric plasma jet (Medaion; Plasma Technologists) with a constant 9 kW voltage difference and 65 kHz frequency at a distance of 1 cm from the tooth for 60 s. Group 4 was treated with flushing of 10 mL of 17% EDTA for 60 s and the atmospheric plasma jet for 60 s. The atmospheric plasma jet was fed constantly with a 9 kW voltage difference and 65 kHz with helium gas.

Immediately after surface treatment, fiber posts (size 2, 1.2 mm; Nordic Glassix) were cleaned with 70% alcohol. The master‐dent resin cement (Dentonics) was prepared according to the manufacturer's instructions and injected directly into the canal. After filling the canal with cement, the fiber posts were placed in the prepared post space. The cement was cured 3 s via light cure with the photoactivation power of 1000 mW/cm^2^ (Woodpecker LED.B; Guilin Woodpecker Medical Instrument Co.). After removing excess cement, an extra 20 s of light cure was applied. Upon filling the canal with cement, the fiber posts were placed in the prepared post space. The posts were bonded by an operator who was well acquainted with the cementing process in accordance with the manufacturer's instructions.

### 2.3 Push‐out test

After keeping the specimen at 37°C and 100% humidity in an incubator (Thermo Scientific™ Heratherm™; Thermo Fisher Scientific Inc.) for 24 h, 1‐mm‐thick disk‐shaped sections from coronal and apical third of the root were cut using a diamond disk (Mecatome; Presi). To consider the same level of the canal at the coronal or apical part, teeth were chosen with the same length and embedded in resin. The thickness of the specimen was measured using a Mitutoyo absolute digital caliper (Mitutoyo Corp.) with an accuracy of 0.001 mm. The diameter of the post on each specimen was measured by a calibrated microscope for push‐out test calculations.

The specimens (dental slices) were placed in the universal testing machine (Zwick Roell; Z020). The diameter of the jig was adjusted to 0.8 mm. The test was performed only by applying force to the fiber posts and not to the resin cement or dentin. The force was applied to the specimens at a speed of 1 mm/min in the apical–coronal direction until the specimens’ failure. The bond strength of each piece was calculated by megapascals using the following formula:

$$\begin{array}{rc} \mathrm{Micro}\;\mathrm{push}‐\mathrm{out}\;\mathrm{bond}\;\mathrm{strength}= & F/A[A=\pi (r_{1}\;+\;r_{2})\\ & \surd (r_{1}\;-\;r_{2})2\;+\;h_{2}], \end{array}$$

where *r*_1_ is the post radius at the coronal level, *r*_2_ the post radius on the other level, and *h* the thickness of each piece.

### 2.4 Evaluation of the failure pattern

The failure pattern was evaluated using a stereo microscope (Nikon Inc.) at ×10 magnification. The type of failure was classified as follows: AD, adhesive failure between dentin and cement (post is entirely covered with resin cement); AP, adhesive failure between cement and post (no cement remaining on the post); CD, cohesive failure in dentin; CP, cohesive failure in the post; M1, mixed failure such that 0%–50% of the post surface was covered with cement; M2, mixed failure such that 50%–100% of the post surface was covered with cement.

Three dental sections were randomly selected from each group for scanning electron microscopy evaluation. The sections of isolated posts and failure areas were then evaluated using a computer and electron microscope (FEI Nova NanoSEM 450; magnification: ×100 and ×1000; Sadeghi Mahounak et al., 2021).

This article's methodology followed the CRIS guidelines (Krithikadatta et al., 2014).

### 2.5 Statistical analysis

In addition, all variables were tested for normal distribution by the Shapiro–Wilk test. An independent *t*‐test was used to compare the bond strength at two root points and the type of surface preparation (EDTA and plasma) was considered the between‐subject factor. Data were entered into IBM SPSS software (version 20.0; SPSS Inc.) and the one‐way analysis of variance statistical test was used to evaluate the effect of different root canal dentin surface treatments on the bond strength of tooth‐colored posts in different areas of the root canal (*p* < .05). The acceptable type I error in this study was equal to 0.05. Table 1 summarizes the descriptive data on the bond strength of fiber post‐to‐root dentin obtained by push‐out test in different areas/groups.

**Table 1:** Descriptive data on bond strength (MPa) of fiber post‐to‐root canal dentin in coronal and apical thirds.

Groups	Minimum bond strength		Maximum bond strength		Mean and SD of bond strength	
	Coronal	Apical	Coronal	Apical	Coronal	Apical
Normal saline	4.24	1.73	6.16	3.94	4.75 ± 0.53	2.44 ± 0.71
EDTA	2.68	1.70	5.50	4.50	4.30 ± 0.88	2.76 ± 0.95
CAP	5.90	5.70	11.14	8.31	9.96 ± 0.83	7.41 ± 0.58
EDTA + CAP	8.13	6.71	19.10	15	13.86 ± 4	9.18 ± 2.38

Abbreviations: CAP, cold atmospheric plasma; EDTA, ethylenediaminetetraacetic acid.

## 3 RESULTS

**Figure 1:**
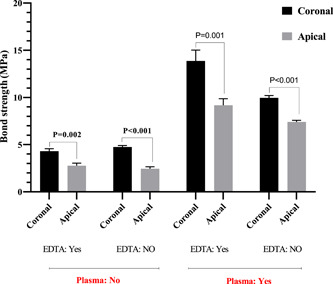
Comparison of the fiber post bond strength to the root canal dentin in the coronal and apical thirds. EDTA, ethylenediaminetetraacetic acid; NO, nitric oxide.

**Figure 2:**
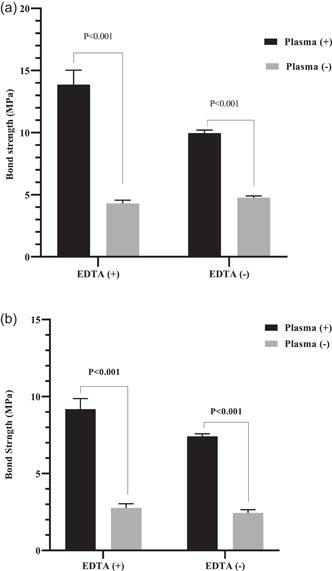
Comparison of the effect of the cold atmospheric plasma (CAP) on increasing the fiber post bond strength to the root canal dentin in the coronal third (a). Comparison of the effect of the CAP on increasing the fiber post bond strength to the root canal dentin in the apical third (b). EDTA, ethylenediaminetetraacetic acid.

The maximum fiber post bond strength to the root canal dentin in the coronal part was observed in the CAP + EDTA group followed by the CAP group. The minimum bond strength was related to the EDTA and control groups in both coronal and apical thirds. The fiber post bond strength was significantly higher in the CAP + EDTA group than in the CAP and EDTA groups (*p* = .003 and *p* < .0001, respectively). Furthermore, significantly higher bond strength was observed in the CAP group than in the control group (*p* < .0001). No significant difference was found in the bond strength between the EDTA and control groups (*p* = .146) and between the CAP group and EDTA (*p* = .076). According to the results of the *t*‐test, the same trend was observed in the apical part. The bond strength was significantly higher in the coronal third than in the apical third in all the groups (*p* < .0001) (Figure 1). The use of CAP increased the fiber post bond strength in both coronal and apical thirds (Figure 2).

### 3.1 Failure pattern results (Table 2 and Figure 3)

**Table 2:** Frequency distribution of the modes of failure in different parts of the root.

Plasma			EDTA		EDTA	
			0	1	0	1
No	Failure	AD	5	5	4	2
		AP	2	2	3	1
		CD	2	1	0	1
		CP	0	2	0	1
		M1	1	1	2	3
		M2	2	1	2	4
	Total		12	12	12	12
Yes	Failure	AD	3	1	1	2
		AP	1	1	2	0
		CD	1	1	0	0
		CP	0	1	2	1
		M1	5	5	4	5
		M2	2	3	3	4
	Total		12	12	12	12
	Root area		Apical		Coronal	

Abbreviations: AD, adhesive failure between dentin and cement (post is entirely covered with resin cement); AP, adhesive failure between cement and post (no cement remaining on the post); CD, cohesive failure in dentin; CP, cohesive failure in the post; EDTA, ethylenediaminetetraacetic acid; M1, mixed failure such that 0%–50% of the post surface was covered with cement; M2, mixed failure such that 50%–100% of the post surface was covered with cement.

**Figure 3:**
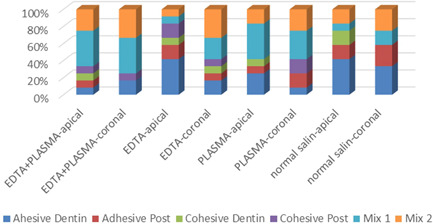
Failure patterns in different groups. EDTA, ethylenediaminetetraacetic acid.

**Figure 4:**
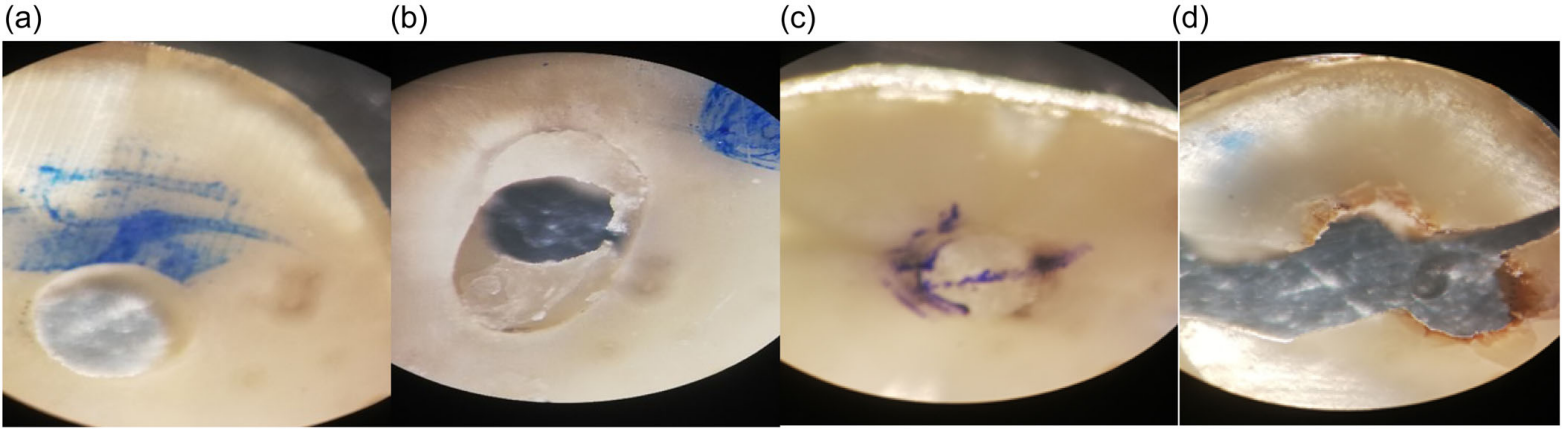
Stereomicroscope results of failure pattern. (a) Adhesive failure between dentin and cement, (b) Adhesive failure between dentin and post, (c) cohesive failure in post, and (d) cohesive failure in dentin.

**Figure 5:**
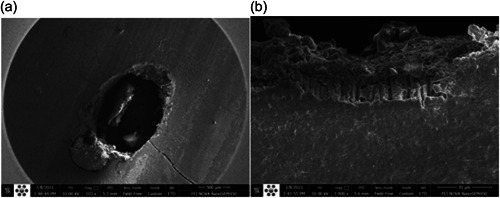
Scanning electron microscope (SEM) results of failure pattern. (a) SEM view of Mix 1 and (b) SEM view of penetration of resin into dentin tubules.

The *χ* ^2^ test did not show a significant difference between different groups in terms of failure patterns in different thirds (*p* > .05). Figures 4 and 5 show the results of the stereomicroscope evaluation.

## 4 DISCUSSION

Posts have been used for many years to restore endodontically treated teeth. Fiber posts have received more attention because of its reduced risk of root failure, better esthetics, higher flexural strength, and lower fatigue. Furthermore, fiber posts have a coefficient of elasticity close to that of dentin and have appropriate power distribution (Jawed et al., 2022). Pull‐out and push‐out methods are used to measure the bond strength between the post and the root canal. The push‐out method is more applicable because it better simulates clinical conditions. Micro push‐out is a modified method and exhibited better results in reducing the thickness of the specimen (Kececi et al., 2008). This study used AH Plus resin sealer, which was previously shown to have no significant adverse effect on resin cement bond strength and the potential to bond to resin cement (Dibaji et al., 2017; Nesello et al., 2022; Reyhani et al., 2016). This study was conducted to evaluate the effect of root dentin surface pretreatment with CAP on improving the bond strength of fiber posts. In the present study, no surface treatments including etching or using sealants were performed because of the negative effect on bond strength (Lamichhane et al., 2014; Sadeghi Mahounak et al., 2021). Also, it has been stated that the weakest interface is between cement and root canal dentin, thus implying that surface treatment of fiber posts did not yield significant outcomes Jawed et al. (2022).

The results exhibited that the CAP increased the fiber post bond strength significantly in both coronal and apical thirds compared to the control and EDTA groups. It is possible that the CAP changes the molecular properties of the dentin surface and also increases dentin surface energy, which subsequently improves the bond strength (Sadeghi Mahounak et al., 2021). The CAP includes highly energetic molecules that can increase surface energy and transfer hydrophilic properties to solids by removing hydrocarbons and forming hydroxyl groups (Zhang et al., 2014). This phenomenon facilitates better penetration of adhesives. The CAP surface treatment potentially opens dentin tubules by chemically converting organic compounds to volatile components, thus removing organic debris from dentin tubules (Dong et al., 2013). The CAP can also induce a chemical bond between 2‐hydroxyethyl methacrylate (HEMA) and dentin (Chen et al., 2014). Reactive oxygen species produced by the CAP can participate in the elevation of hydrogen bonds between collagen fibers and adhesives by generating carbonyl groups in type I collagen molecules through surface interactions (Huang et al., 2007). Strong chemical and physical bonds resulting from the CAP subsequently cause the migration of the HEMA to the demineralized dentin, which leads to better penetration of the resin into the dentinal tubules.

Few studies have evaluated the effect of CAP on bond strength (Stancampiano et al., 2019; Yeter et al., 2020). Prado et al. (2016) concluded that argon plasma treatment increases the penetration of resin‐based sealer into the dentin in the coronal third. The type of plasma, duration of use, and distance of the plasma jet nozzle from the plasma‐prepared surface affect the bond strength of the resin. The present study shows that the CAP irradiation for 60 s has a significant effect on bond strength, unlike the results of Mahounak et al., which had a plasma irradiation parameter of 180 s. The distance between the plasma nozzle and the tooth was 1 cm in the present study and was 2 cm in the Mahounak study as longer distances can result in energetic gas molecules deviating around the instrument and escaping, thereby diminishing the effectiveness of the treatment (Sadeghi Mahounak et al., 2021). This difference appears to reduce the penetration of the CAP into the root canal and also explains the rationale for decreasing the time of plasma usage in this study. Another study depicted that increasing the plasma irradiation time (45–60 s) reduces collagen fibers, which reduces the bond strength (Zhu et al., 2018). Therefore, various parameters such as plasma contact time, plasma jet distance from the tooth, and the potential difference can result in different outcomes (Sadeghi Mahounak et al., 2021).

Concomitant use of the CAP + EDTA in this study showed a significantly higher bond strength than the CAP group. However, the bond strength values in this group were significantly higher than the EDTA and control groups. The synergistic effect of the CAP with EDTA increased the surface energy of dentin tubules and the bond strength (Zhu et al., 2018). it appears that the use of a chelating agent before using the CAP can increase the possibility of plasma interfering with the dentin surface by decreasing the smear layer. Therefore, using chelating agents such as EDTA can remove the smear layer during the cleaning step (Kong et al., 2015; Ozkocak & Sonat, 2015). Treatment with EDTA and the CAP increases the wettability of dentin and the permeability of adhesive compounds which subsequently increases the bond strength.

In general, the bond strength values in all coronal third groups were higher than the apical third groups. This can be related to CAP's better penetration. Furthermore, the lack of proper access of the plasma gas nozzle to the apical portion, inadequate polymerization, less excitation of surface molecules, more complex canal geometry, presence of subcanals, and increased *c* factor lead to the lower bond strength in the apical third (Sadeghi Mahounak et al., 2021). The coronal third of the root, in comparison, has cleaner, larger, denser, and vertically oriented dentin tubules. These results are consistent with the study by Alkhudhairy et al. (2018) and Alaghemand et al. (2013) Moreover, the presence of water affects the function of CAP by preventing dehydration of the dentin surface caused by CAP and collagen collapse and induces better resin penetration around the collagen fibers (Dong et al., 2014).

As a weak acid, EDTA can remove the sealer and smear a layer on the root canal without reducing the mineral content of dentin. This mineral content is needed for resin cement chemical bonding. The chelation of calcium in root dentin occurs with the phosphate ester in the resin cement and improves the micromechanical bond. However, increasing the EDTA exposure can remove large amounts of calcium ions from the dentin and consequently destroys bonding (Barreto et al., 2016; Ramírez‐Bommer et al., 2018).

This study showed that the use of EDTA did not cause a significant difference in the bond strength compared to the control group. This is in agreement with the study of Garcia et al. (2018) and Barreto et al. (2016), but does not align with the studies of Gu et al. (2009) and Sadeghi Mahounak et al. (2021). The different concentrations of EDTA and the different types of resin cements used can explain these differences.

One of the limitations of the present study includes gas penetration and transmission into the canal because there was a lack of a special thin tip for transferring the plasma gas into the canal. Also, the high cost of using helium plasma gas was another limitation because of the high cost of the gas itself and the technology, which were used to convert it to plasma gas. Finally, the effect of different CAP duration, different doses of EDTA, and other chelating agents on bond strength are encouraged to be investigated in future studies.

## 5 CONCLUSION

With the limitations of the present study, it can be concluded that the use of CAP alone and the use of CAP in conjunction with EDTA can both have significant positive effects (*p* < .001) on increasing the bond strength of fiber posts to the root canal dentin.

## AUTHOR CONTRIBUTIONS

*Conceptualization*: Sara Valizadeh. *Methodology*: Neshatafarin Manouchehri, Dorsa Seyedi. *Formal analysis*: Neshatafarin Manouchehri, Sara Valizadeh. *Writing—original draft preparation*: Neshatafarin Manouchehri. *Writing—review and editing*: Sara Valizadeh, Safoura Ghodsi, and Faezeh Atri. *Supervision*: Sara Valizadeh, Safoura Ghodsi, and Faezeh Atri. All authors have read and agreed to the published version of the manuscript.

## CONFLICT OF INTEREST STATEMENT

The authors declare no conflict of interest.

## Data Availability

The data regarding this research article will be provided upon formal request from the corresponding author.
